# Addressing disparities in oral health access and outcomes for aging adults in the United States

**DOI:** 10.3389/fdmed.2025.1522892

**Published:** 2025-04-01

**Authors:** Adam Lowenstein, Mabi L. Singh, Athena S. Papas

**Affiliations:** ^1^ Department of Basic and Clinical Translational Sciences, Tufts University School of Dental Medicine, Boston, MA, United States; ^2^ Division Oral Medicine, Department of Diagnostic Sciences, Tufts University School of Dental Medicine, Boston, MA, United States

**Keywords:** aged, oral health, healthcare disparities, dental care for aged, health literacy, socioeconomic factors, periodontal diseases, dental caries

## Abstract

Oral health is essential for the overall well-being of aging adults, yet significant disparities persist in the United States, contributing to malnutrition, reduced quality of life, and social isolation. Despite advancements in preventive dentistry, older adults continue to face substantial oral health challenges. This study reviewed the current state of oral health among aging adults in the United States, analyzing national health surveys and existing preventive dentistry protocols to assess their effectiveness. National data indicated that a high percentage of older adults experience tooth loss, periodontal disease, and root caries. Results indicated that almost 40% of adults aged 65 and older have lost a significant number of teeth, and a large proportion of the aging adult population has suffered from high rates of caries and periodontal disease. Socioeconomic factors were found to significantly influence access to dental care. Key barriers included low income, inadequate insurance, and low oral health literacy exacerbating disparities. The study proposes a multifaceted approach to improve oral health equity, including policy changes, community-based preventive programs, enhanced geriatric dentistry education, and the integration of oral health into primary care. Addressing these disparities is needed to improve both oral and overall health outcomes for the growing elderly population in the United States.

## Introduction

Oral health is critical for overall health and quality of life, particularly for aging adults who are vulnerable to systemic health issues exacerbated by poor oral conditions. Chronic oral diseases, including tooth loss, periodontal disease, and untreated dental caries, are prevalent among adults aged 65 and older and are correlated with malnutrition, cognitive decline, cardiovascular disease, and other systemic conditions (1, 2). Studies indicate that tooth loss and poor oral health significantly impact dietary intake, limiting the ability to consume nutrient-rich foods such as fruits, vegetables, and lean proteins, which are essential for managing chronic diseases and supporting immune function (3–14). According to the U.S. Census Bureau, the number of adults aged 65 and older is projected to reach 98 million by 2060, underscoring the urgent need for effective interventions to address disparities in access and outcomes in oral healthcare (15).

Despite advancements in preventive dental practices, access remains inequitable. Vulnerable groups – including low-income, minority, and rural populations – face barriers like inadequate insurance, low oral health literacy, and limited local access to care. These disparities contribute to higher rates of caries and edentulism, creating a cycle of health inequity. Previous research has shown that these populations also experience a high prevalence of polypharmacy, leading to side effects such as xerostomia, which can further exacerbate caries risk. Given the interdependence of oral and systemic health, addressing these disparities is essential not only for oral health but also for overall health and well-being in aging adults (3, 4, 6, 7, 12, 16).

This study aims to review the literature and provide a comprehensive discussion on the evidence related to disparities in oral health access and outcomes among adults aged 65 and older in the United States. Additionally, we discuss the role of health literacy in this context and the consequences associated with oral health disorders.

The analysis emphasized the following three main areas: demographic disparities in access to health services and outcomes in oral health, health literacy, and the consequences of disparities in oral health. There was an examination of (i) differences in oral health outcomes by income, race, and insurance status, identifying populations most vulnerable to poor oral health outcomes; (ii) analysis of the role of oral health literacy in shaping preventive care behaviors and health outcomes, drawing on data from health literacy studies specific to older adults; (iii) the consequences of oral diseases, for example on the nutritional intake among adults and the relationship between oral health and chronic diseases, such as diabetes and cardiovascular disease, highlighting the implications of oral health disparities on broader health outcomes.

## Demographic disparities

National Health and Nutrition Examination Survey data indicated that approximately 40% of adults aged 65 and older experience significant tooth loss, with about 13% who have lost all of their teeth (17). These issues were most prevalent among low socioeconomic and minority populations, particularly African American and Hispanic individuals (18). This tooth loss was shown to significantly limit dietary options, reducing intake of key nutrients that are more difficult to consume without functional dentition.

Socioeconomic and geographic factors further compounded these disparities. National data revealed that older adults with household incomes below the federal poverty level had more untreated dental caries compared to those with higher incomes (19). Rural older adults faced a significantly higher prevalence of tooth loss and untreated decay, with the gap pronounced in states that lack adequate dental workforce distribution (20). These systemic barriers contributed to persistent oral health inequities, especially affecting racial and ethnic minority communities who historically faced discrimination in healthcare access.

The prevalence of untreated caries and periodontal disease was high across the elderly population, with nearly 96% of older adults experiencing some degree of dental caries (21). Disparities were most pronounced among those with limited financial resources and inadequate insurance coverage. Periodontal disease and root caries presented higher risks for aging adults with chronic illnesses, notably diabetes, underscoring the importance of integrated care approaches.

## Health literacy

Oral health literacy levels were notably low among older adults in low-income and minority populations, which limited their understanding of preventive care practices. This was directly correlated with lower utilization rates of dental services, as individuals with limited literacy in oral health were less likely to prioritize preventive care. This lack of preventive care contributes to the observed disparities in oral health outcomes (22).

Public education and health literacy campaigns would be beneficial as well. National public health campaigns to improve oral health literacy among aging adults and caregivers could facilitate better understanding and utilization of preventive practices. Emphasis on accessible information about the importance of regular dental visits, dietary considerations, and proper denture use would empower aging adults to make informed choices regarding their oral health. Programs targeting caregivers, particularly in long-term care settings, could focus on daily oral hygiene practices and the nutritional needs of older adults, thereby reducing preventable oral health issues and supporting systemic health (23).

## Nutritional correlations

**Figure 1:**
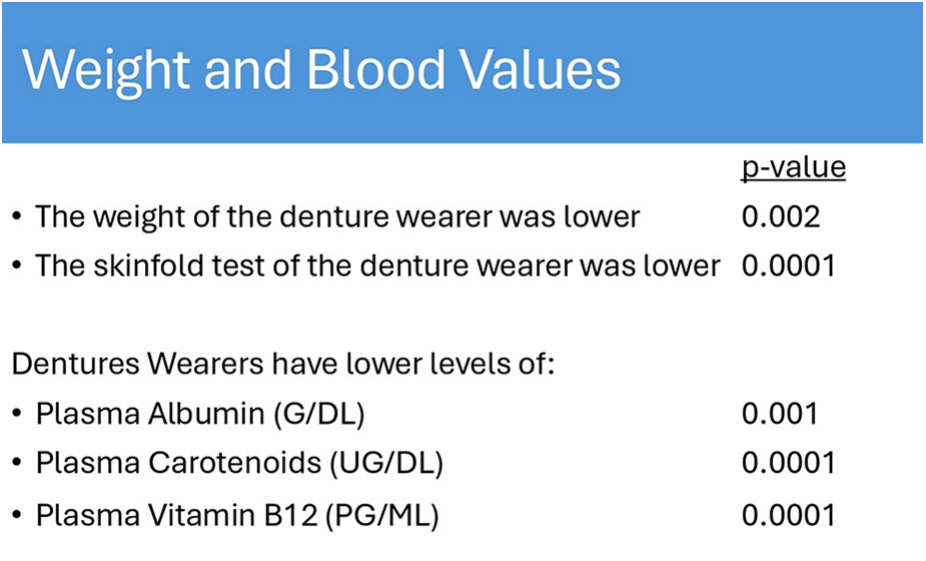
Fiber consumption from 3-Day food diaries (table adapted from Papas et al., 199c8).

**Figure 2:**
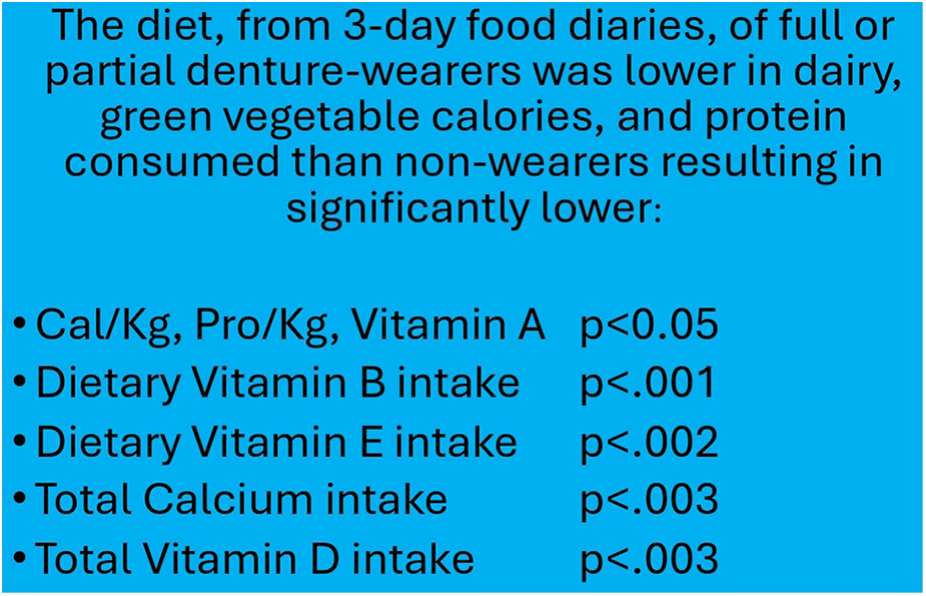
Weight and blood values of denture wearers (table adapted from Papas et al., 1998).

**Figure 3:**
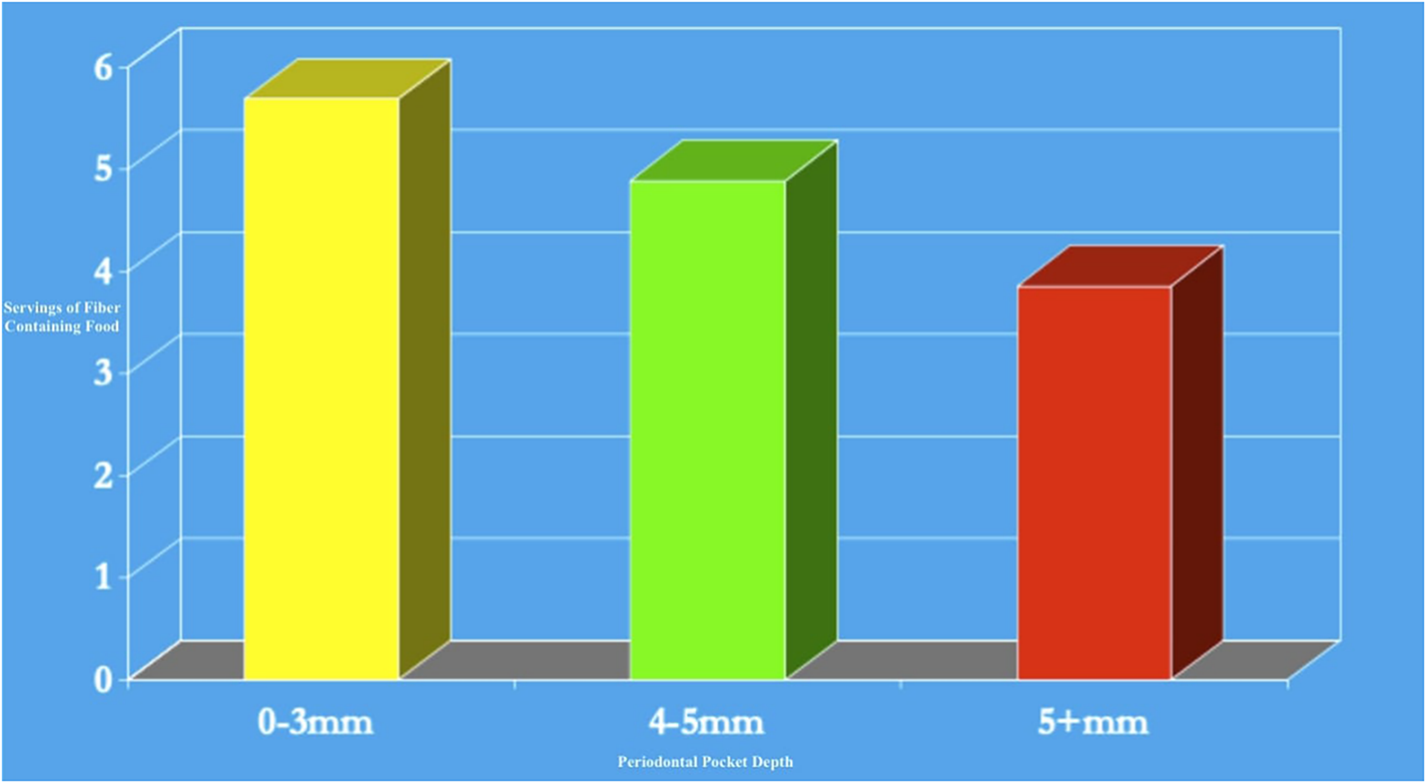
Dairy, green vegetable calories, and protein consumed in denture wearers compared to non-wearers (table adapted from Papas et al., 1998).

**Figure 4:**
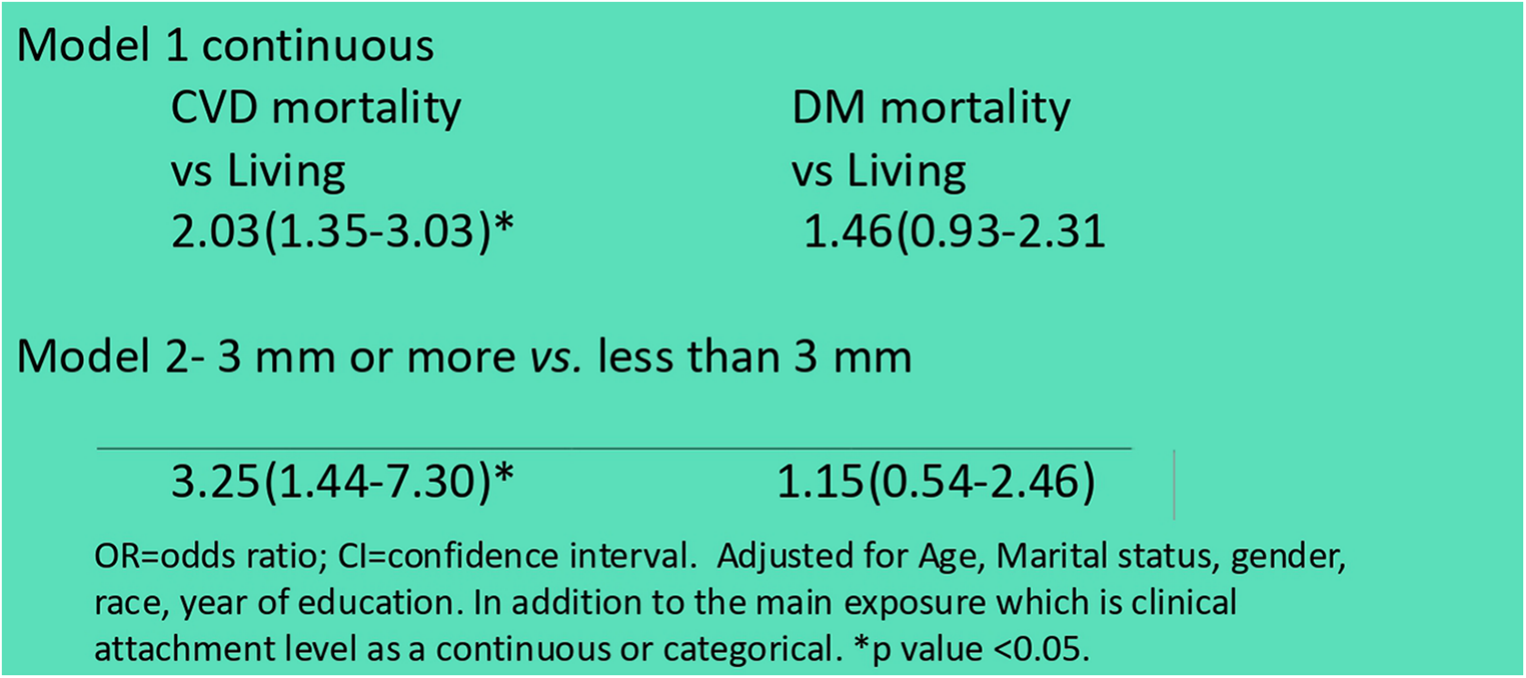
Association of CAL with mortality (table adapted from Natto et al., 2015).

The analysis revealed that poor oral health directly impacted dietary choices, reducing intake of high-fiber fruits and vegetables, lean proteins, and other nutrient-dense foods. Deficiencies in these essential nutrients may hasten Alzheimer's disease, Parkinson's disease, cognitive function, and systemic health problems such as cardiovascular disease, arterial inflammation, and diabetes, contributing to a downward spiral in both oral and systemic health (24–28). In addition, 88% of persons over the age of 60 are taking one or more medications (29). Additionally, the side effects of common medications taken by older adults, such as dry mouth, compounded these issues by increasing susceptibility to caries and other oral infections as well as difficulty speaking and swallowing (30, 31). Dry mouth, or hyposalivation, is a frequent side-effect of consuming multiple medicines, dehydration, diabetes, Sjögren's Syndrome, radiation, and chemotherapy (32). Periodontal disease causes individuals to decrease fiber intake in their diet, and this phenomenon is similar to those with partial or full dentures, as described in Figures 1–3 (8, 33, 59). Our previous research also indicated an association between clinical attachment level (CAL) and mortality. We found that individuals with increased CAL were more than twice as likely to have mortality due to cardiovascular disease than those with normal CAL, as shown in Figure 4 (10).

Research demonstrates that functional teeth and overall nutritional status are closely interconnected in older Americans (34). Nutritional status impacts oral health, while oral health directly affects one's ability to maintain proper nutrition (35, 36). For aging adults, a lifetime of poor dietary choices, insufficient oral hygiene, and untreated gingivitis can culminate in tooth pain, tooth loss, and inflamed, bleeding gums. These oral health problems create significant barriers to consuming nutritious foods like fresh fruits and vegetables, whole grains, lean meats, and hard cheeses, ultimately compromising both nutrient and caloric intake.

Older Americans who struggle with chewing and swallowing frequently show insufficient intake of essential nutrients, including fiber, linoleic acid, and various minerals such as potassium, calcium, magnesium, zinc, and selenium (37). They also often lack adequate levels of vitamins D, E, and K, as well as folate, biotin, and molybdenum (38). Studies have found that older adults without natural teeth or those who rely on dentures consume significantly fewer fruits and vegetables, particularly those rich in carotenoids and vitamin C, compared to those with natural teeth, as seen in Figure 2 (8, 33, 39, 40, 59). Instead, these individuals tend to favor carbohydrate-rich foods that are easier to chew, potentially worsening both their oral health and overall well-being.

Research has revealed that the number of natural teeth is inversely related to several health metrics, including BMI, waist circumference, blood pressure, and fasting blood glucose (41). Those who wear dentures frequently experience involuntary weight loss and frailty, as described in Figure 1 (8, 33, 42, 59). Scientific reviews have documented the complex pathophysiological pathways linking sarcopenia, dysphagia, and oral health, all of which contribute to increased frailty in older adults (43).

Appropriate dietary changes and improved oral hygiene habits can help individuals maintain healthier teeth, support a beneficial oral microbiome, and reverse the common trend toward tooth loss, decay, and gum disease (44). A 2018 systematic review of 26 studies found that well-nourished individuals had significantly more functional tooth units compared to those at risk of malnutrition or who were malnourished (36). This further reinforces the bidirectional connection between diet and oral health.

The frequent presence of fermentable carbohydrates – sugars and starches – in the mouth produces a greater cariogenic effect than the absolute quantity of carbohydrates consumed. Extended oral exposure to carbohydrates enhances the production of bacterial acids and microbial biofilm, does not allow saliva to neutralize the acids, and eventually leads to enamel demineralization (45–47). In periodontitis, the sugars also drive oxidative stress and may trigger a hyper-inflammatory state. To help clean teeth and stimulate saliva after meals and snacks, aging adults should brush their teeth with fluoridated toothpaste, floss and clean between teeth, or chew sugar-free gum (48). Research suggests that chewing sugar-free gum can improve salivary flow rates, reduce new dental caries by 28%, and lower oral biofilm and Streptococcus mutans levels (49, 50). While not a replacement for good dental hygiene, sugar-free gum can have a significant positive impact on dental health.

These nutritional impacts are particularly severe among socioeconomically disadvantaged populations. Among racial and ethnic minorities, the combination of poor oral health and limited access to nutritious foods creates a compound effect – African American and Hispanic older adults with untreated dental disease show lower consumption of fresh fruits and vegetables compared to white counterparts with similar oral health status (51). Rural elderly populations face additional challenges, as both limited dental access and food deserts contribute to poorer nutritional outcomes. In addition, inner-city poor individuals consumed more sugar and refined carbohydrates and had more oral disease. The inability to afford dental care often forces these vulnerable populations to rely on softer, more processed foods, and this phenomenon creates a cycle of declining oral and systemic health (52).

A combined approach of dietary improvements and enhanced oral hygiene can help older adults maintain healthier teeth, support a beneficial oral microbiome, and improve their overall nutritional status. Addressing these interconnected factors is important for promoting healthy aging and preventing the negative spiral of poor oral health leading to inadequate nutrition.

## Discussion

The findings highlight multiple interconnected factors driving oral health disparities among aging adults and underscore the need for targeted policy and healthcare interventions. Key recommendations include policy reforms to expand access. Expanding Medicare coverage to include routine dental services would address a substantial financial barrier for many aging adults, particularly those on fixed incomes. Medicaid expansions in states with limited dental benefits would also reduce disparities in underserved populations. Increased federal funding for oral health programs targeting aging adults, especially in low-income and rural areas, could enhance access to care and preventive services.

In addition, preventive and community-based programs would be advantageous. Community-based mobile dental clinics could improve access to care in rural areas, providing preventive services and screenings to older adults who otherwise lack access to routine dental care (41, 53). This is particularly valuable for those with mobility issues or transportation limitations. Expanding fluoride access in rural areas could prevent caries, reducing dental disease by approximately 25% among those exposed (54). A fluoridation program at this scale could result in significant cost savings, with an estimated $20 return on investment per $1 spent on water fluoridation, benefiting entire communities (55). Medical and dental curricula should emphasize geriatric dentistry, with a focus on preventive care, management of polypharmacy effects, and treatment of age-specific conditions. Provider training should also cover the nutritional impacts of oral health and the specific needs of elderly patients, particularly in underserved communities.

Given the link between oral health and systemic conditions, routine primary care visits for aging adults should include an oral health assessment. Primary care providers should receive training in basic oral health screening and counseling to reinforce preventive behaviors and facilitate timely referrals to dental professionals.

The inclusion of oral health-related dietary guidelines in the 2025–2030 Dietary Guidelines for Americans could further emphasize the importance of nutrient-dense, easy-to-chew foods, helping to mitigate the impact of poor oral health on diet and nutrition (3, 10, 56, 57). Recommendations would focus on foods high in fiber, vitamins, and minerals, critical for older adults' health. Efforts to promote soft-textured, nutrient-dense options could improve dietary intake among those with compromised oral function.

Initiatives have been implemented to address oral health inequalities in the United States. The Health Resources and Services Administration's Grants to States to Support Oral Health Workforce Activities program showed promise in expanding access to care in underserved areas, with greater access to oral healthcare in the Midwest and West (58). Initiatives like this one highlight both the potential for successful interventions and the ongoing challenges in addressing oral health inequalities systematically. More sustainable funding and broader policy support remain critical barriers to achieving comprehensive improvements in oral health equity.

## Conclusion

This study underscores the substantial oral health challenges facing aging adults in the United States, particularly those in socioeconomically disadvantaged groups. The proposed recommendations – ranging from policy and insurance reforms to community-based programs and health literacy improvements – offer a framework to reduce disparities and improve outcomes for aging adults. An integrated approach addressing both preventive and therapeutic needs can enhance oral health and, consequently, overall health and quality of life for older adults. These strategies, if implemented, could foster a more equitable healthcare system, ensuring that all aging adults have access to essential oral health services and nutrition support.
